# Supplementary material for: Content-rich biological network constructed by mining PubMed abstracts
Source: BMC Bioinformatics. 2004 Oct 8;5:147. doi: 10.1186/1471-2105-5-147 (PMC528731; doi:10.1186/1471-2105-5-147)
Supplement: Additional File 2 — The original results of the above study (non-essential files are deleted to keep the file size under the limit set by BMC bioinformatics). [file 1471-2105-5-147-S2.bz2 › chilibotAdditionalFile2/dip40/479ID8702798E10568/html/IL8RB_CXCL2.html]

 


 **IL8RB** and **CXCL2** 
  
Found 50 abstracts in PubMed, retrieved 40.  
 

 What does Google say? 
 PDF only 
| .edu only 

---

**Interactive relationship** (e.g. stimulation, inhibition, etc)

**Neutral relationship**- Together these experiments support the notion that increased expression of KC CXCL1 and MIP 2  [ **CXCL2** ]   **CXCL2**  3 and their interaction with CXCR2  [ **IL8RB** ]  are important in the pathogeneses of VILI.  Ref: 12464676 J Clin Invest, 2002
- Astrocytes from CXCR2  [ **IL8RB** ]  deficient mice still respond to KC and MIP 2  [ **CXCL2** ] , indicating the presence of an alternative or novel high affinity receptor for these ligands.  Ref: 11034412 J Immunol, 2000
- Moreover, a significant decrease in interleukin 12 IL 12 levels, in contrast to the increases in KC, MIP 2  [ **CXCL2** ] , and LIX levels, was demonstrated for CXCR2  [ **IL8RB** ]  blocked mice.  Ref: 11254553 Infect Immun, 2001
- Studies presented herein indicate that the CXCR2  [ **IL8RB** ]  chemokine receptor that interacts with selective chemokine ligands, particularly GRObeta  **CXCL2**  and GRObeta T, may be the dominant receptor mediating hematopoietic cell mobilization,.  Ref: 12270782 Crit Rev Oncol Hematol, 2002
- One such receptor, CXCR2  [ **IL8RB** ] , binds multiple CXC chemokines, including interleukin 8, GRO alpha, GRO beta  [ **CXCL2** ] , GRO gamma, and NAP 2.  Ref: 10747307 Arch Pathol Lab Med, 2000
- The CXCR2  [ **IL8RB** ]  is a GPCR that is activated, amongst the others, by the chemokines CXCL8 IL 8 and  **CXCL2**  GROb  [ **CXCL2** ]  to induce cell chemotaxis.  Ref: 12888558 J Biol ChemJ Biol Chem, 2000
- CXC chemokines, macrophage inflammatory protein 2  [ **CXCL2** ]  MIP 2  [ **CXCL2** ]  and KC, a cloning designation based on ordinate and abscissa position as well as the CXC chemokine receptor, CXCR2  [ **IL8RB** ] , are expressed in a variety of cells and tissues in adult mice.  Ref: 11444905 Cytokine, 2001

**Non-interactive relationship** (e.g. studied together, co-existance, homology, etc.)

- Taken together, these data demonstrate that Hem induced priming of PMN not only mediates ALI in the mouse, but also that this process is differentially effected by MIP2  [ **CXCL2** ]  and KC, despite the fact that both signal through CXCR2  [ **IL8RB** ] .  Ref: 12688548 Shock, 2003
- These studies demonstrate the importance of the CXCR2  [ **IL8RB** ]  ligands MIP 2  [ **CXCL2** ]  and KC and neutrophils in the acute host response to S. aureus in the brain.  Ref: 11254722 J Immunol, 2001
- The expression of alpha amino 3 hydroxy 5 methyl 4 isoxazolepropionate AMPA type glutamate receptor 1 GluR1 greatly impairs the chemotaxis induced by  **CXCL2**  in CXCR2  [ **IL8RB** ]  expressing HEK cells.  Ref: 12507773 J Neuroimmunol, 2003
- During the development of nephrotoxic nephritis NTN in the mouse, we find that a variety of chemokines and chemokine receptors are induced CCR1 RANTES, MIP 1alpha, CCR2 MCP 1, CCR5 RANTES, MIP 1alpha, MIP 1beta, CXCR2  [ **IL8RB** ]  MIP 2  [ **CXCL2** ] , and CXCR3 IP 10.  Ref: 10587518 J Clin Invest, 1999
- In the first week, mast cell degranulation and leukocyte influx mononuclear cell, MNC, and polymorphonuclear cell, PMN were associated with CXCR2  [ **IL8RB** ] , KC and macrophage inflammatory protein MIP 2  [ **CXCL2** ]  mRNA expression, as determined by TaqMan reverse transcriptase polymerase chain reaction.  Ref: 11704636 Br J Pharmacol, 2001
- The results indicate that rat CXCR2  [ **IL8RB** ]  on neutrophils is the unique receptor for all three types of CINCs, and CINC 1 2 and CINC 3  [ **CXCL2** ]  exert different biological activities through the common receptor.  Ref: 10975996 Cytokine, 2000
- For example strong calcium responses were seen in neutrophils following stimulation with the CXCR1 and CXCR2  [ **IL8RB** ]  ligands, interleukin IL 8, GCP 2 and Gro beta  [ **CXCL2** ] .  Ref: 11298490 Cytokine, 2001
- However, KC and Duffy were less prevalent in the developing cardiovascular system, lung and other organs, muscle and bone, than are CXCR2  [ **IL8RB** ]  and MIP 2  [ **CXCL2** ] .  Ref: 11444905 Cytokine, 2001
- We observed strong correlation between the expression of MIP 2  [ **CXCL2** ]  and CXCR2  [ **IL8RB** ]  in the developing brain, cardiovascular system and condensing mesenchyme between 11.5 and 13.5 days.  Ref: 11444905 Cytokine, 2001
- UTI induced increases in the renal concentrations of the CXCR2  [ **IL8RB** ]  ligands macrophage inflammatory protein 2  [ **CXCL2** ]  and KC, which were not influenced by anti CXCR2  [ **IL8RB** ]  administration.  Ref: 11443555 J Infect Dis, 2001
- CINC 1, CINC 2 and CINC 3  [ **CXCL2** ]  induced calcium mobilizations dose dependently in CXCR2  [ **IL8RB** ]  transfected cells.  Ref: 10975996 Cytokine, 2000
- MIP 2  [ **CXCL2** ]  and CXCR2  [ **IL8RB** ]  are highly expressed in the brain, first in the cerebellum and in the head mesenchyme, the meninges and the floor plate, and by 14.5 days are also present in the telencephalon, thalamus and hypothalamus.  Ref: 11444905 Cytokine, 2001
- Similar to human IL 8 and GCP 2, murine GCP 2 9 78 and macrophage inflammatory protein 2  [ **CXCL2** ]  MIP 2  [ **CXCL2** ]  induced calcium increases in both CXCR1 and CXCR2  [ **IL8RB** ]  transfectants.  Ref: 10570306 J Immunol, 1999
- The release of sICAM 1 was not influenced by IL 8 or MIP 2  [ **CXCL2** ] , although astrocytes and MVEC expressed the IL 8 MIP 2  [ **CXCL2** ]  receptor CXCR 2  [ **IL8RB** ]  as determined by FACS analysis.  Ref: 10861785 J Neurosci Res, 2000
- Further, inhibition of the MIP 2  [ **CXCL2** ]  receptor, CXCR2  [ **IL8RB** ] , decreased baseline hepatocyte proliferation and stat 3 expression in the setting of partial hepatectomy.  Ref: 12875976 Am J Pathol, 2003
- Angiostatin blocked chemotaxis of neutrophils to CXCR2  [ **IL8RB** ]  chemokine receptor agonists IL 8, MIP 2  [ **CXCL2** ] , and GROalpha, formyl Met Leu Phe fMLP, and 12 O tetradecanoylphorbol 13 acetate, and repressed fMLP induced mitochondrial activity.  Ref: 11772950 FASEB J, 2002
- They showed chemotactic activity to MIP 2  [ **CXCL2** ]  in the Boyden chambers and expressed MIP 2  [ **CXCL2** ]  receptor CXCR 2  [ **IL8RB** ]  mRNA confirmed by reverse transcription polymerase chain reaction.  Ref: 11559650 Gut, 2001
- and iii GluR1 binding site cooperativity upon CXCR2  [ **IL8RB** ]  stimulation with CXC chemokine ligand 2  [ **CXCL2** ]   **CXCL2** .  Ref: 12161022 J Neuroimmunol, 2002
- In contrast, the blockade of CXC chemokine receptor 2 CXCR2  [ **IL8RB** ] , a receptor for CXC chemokines, including KC and MIP 2  [ **CXCL2** ] , strikingly enhanced mortality.  Ref: 11254553 Infect Immun, 2001
- Taken together, these results suggest that the association between CXCR2  [ **IL8RB** ]  and AMPARs results in the inhibition of  **CXCL2**  dependent chemotaxis, and may represent a molecular mechanism underlying the modulation of nerve cell migration.  Ref: 12507773 J Neuroimmunol, 2003
- In summary, we have shown that TII alveolar epithelial cells produce three of the major proinflammatory CXC chemokines, GRO, CINC 2alpha and MIP 2  [ **CXCL2** ] , and their cognate receptor CXCR2  [ **IL8RB** ] .  Ref: 12829448 Am J Respir Cell Mol Biol< MedlineTA>Am J Respir Cell Mol Biol, 2003
- In an attempt to develop insight into additional possible subtle roles of CXCR2  [ **IL8RB** ]  and its ligands in the development of the mouse, we examined the expression of MIP 2  [ **CXCL2** ] , KC, CXCR2  [ **IL8RB** ] , as well as the Duffy antigen binding protein for chemokines during embryonic p.c. days 11.5 through 14.5 in the mouse.  Ref: 11444905 Cytokine, 2001
- The expression of the chemokines macrophage inflammatory protein MIP 2  [ **CXCL2** ]  and MIP 1alpha and of their receptors CXCR2  [ **IL8RB** ]  and CCR5 was assessed in wild type WT and TNF lymphotoxin alpha knockout TNF LT alpha mice subjected to closed head injury CHI.  Ref: 11435946 Neuroreport, 2001
- Because the murine CXC chemokines KC CXCL1 and MIP 2  [ **CXCL2** ]   **CXCL2**  3, via CXCR2  [ **IL8RB** ] , are potent neutrophil chemoattractants, we investigated their role in a murine model of VILI.  Ref: 12464676 J Clin Invest, 2002
- MIP 2  [ **CXCL2** ]  neutralization had no detrimental effects on survival, neutrophil recruitment, or bacterial clearance, suggesting the usage of additional or alternative CXCR2  [ **IL8RB** ]  binding ligands.  Ref: 10623839 J Immunol, 2000
- These differences were not due to impaired KC and MIP 2  [ **CXCL2** ]  production in the corneal stroma of CXCR2  [ **IL8RB** ]  mice, which was similar to BALB c mice.  Ref: 11238651 J Immunol, 2001
- In the absence of the high affinity receptor for MIP 2  [ **CXCL2** ]  as in CXCR2  [ **IL8RB** ]  deficient mice or in the presence of a blocking Ab to MIP 2  [ **CXCL2** ] , peripheral tolerance is prevented, and Ag specific T regulatory cells are not generated.  Ref: 11123307 J Immunol, 2001
